# Enhancing Hot Air Drying Efficiency through Electrostatic Field–Ultrasonic Coupling Pretreatment

**DOI:** 10.3390/foods12081727

**Published:** 2023-04-21

**Authors:** Ri-Fu Yang, Ying-Ying Peng, Yu-Rong Wang

**Affiliations:** School of Physics and Optoelectronics, South China University of Technology, Guangzhou 510640, China

**Keywords:** electrostatic field, ultrasonic, coupling pretreatment, ginkgo fruits, drying efficiency

## Abstract

The drying of compact and biologically active materials presents significant challenges. In this study, we propose using electrostatic field–ultrasonic coupling pretreatment to enhance the drying efficiency of ginkgo fruits. We designed and constructed an experimental device to investigate the effects of ultrasonic power, pretreatment time, hot air drying temperature, and electrostatic field voltage on the moisture content of the fruits. We used the response surface methodology to identify optimal process conditions and further explored the kinetic model for the moisture content of the fruits under the pretreatment. The results showed that the optimal process parameters for electrostatic–ultrasound pretreatment and the drying of ginkgo fruits were: an electrostatic field voltage of 11.252 kV, an ultrasound power of 590.074 W, a pretreatment time of 32.799 min, and a hot air drying temperature of 85 °C. Under the optimized process conditions, the correlation between the moisture content of ginkgo fruits and the two-term drying kinetics model was the highest. After electrostatic–ultrasound coupling pretreatment, the drying rate of ginkgo fruits was significantly improved during hot air drying.

## 1. Introduction

Ginkgo fruit, which is commonly known as “Baiguo” in China, hasits production concentrated in Asian countries such as China, Japan, and Korea, with China being the largest producer, accounting for over 90% of global production [1]. Ginkgo fruit is rich in nutrients and potent medicinal ingredients and therefore provides nutritional and health benefits. As an ingredient, it is mainly added to desserts, tonic soups, and dishes, and is also recorded in the Materia Medica as a source of medicine used throughout Chinese history [2]. In addition, the flavonoids, terpene lactones, and polysaccharides in ginkgo fruits have anti-inflammatory and anti-tumor properties [3], as well as containing ginkgolic acid, which has anti-cancer, anti-parasitic, and anti-bacterial properties [4]. Due to the high water content of freshly harvested ginkgo fruit, which exceeds 50%, the nutrients inside the fruit are easily lost or even rot, making it difficult to store. Thus, the fruits require drying to preserve them. Drying methods for ginkgo fruits include natural drying, hot air drying, infrared radiation drying, and microwave drying [5]. Hot air drying is the most popular industrial method due to its simplicity, low cost, and ability to process large quantities. However, prolonged exposure to high temperatures can result in color loss and nutrient depletion [6]. Consequently, strategies to effectively reduce drying time and lower drying temperature are essential [7].

Ultrasound is a widely used method for drying pretreatment due to its mechanical and cavitation effects, which can shorten drying time and reduce temperature [8]. Ultrasonic energy is propagated by means of liquid media (usually water) to make the material structure loose and porous for the subsequent drying process (such as hot air drying, or far infrared drying). Moreira Azoubel et al. used ethanol combined with ultrasound to reduce the drying time of pineapple and obtained an effectively higher moisture diffusion coefficient [9]. Taner Baysal et al. found that pretreatment increased the drying rate of mushrooms by about 37.10% by studying the effects of electrolysis and ultrasound pretreatment, as well as their combination, on the drying rate and quality characteristics of mushrooms [10]. However, the power intensity of existing power ultrasound equipment devices is constrained by the number of ultrasound probes, making it difficult to achieve the desired treatment effect. To address this problem, researchers are exploring new applications of high-power ultrasound, including the introduction of electric fields into coupled ultrasound action. The horizontal component of the electric field force makes the bubble elongate in the direction of field strength, which makes the fluid motion inside the bubble intensify and can significantly improve the ultrasonic cavitation effect. Sobotka J’s experiments demonstrated the potential benefits of direct current electric fields in improving ultrasound cavitation [11]. This paper investigates the effect of electrostatic field and ultrasonic pretreatment (EU-pretreatment) on the drying rate of ginkgo fruits during hot air drying and compares the results with those of ultrasonic pretreatment (U-pretreatment). Few studies in this area have been reported, so it is necessary to investigate the effect of the electric field on drying efficiency.

## 2. Materials and Methods

### 2.1. Experimental Setup and Materials

**Figure 1:**
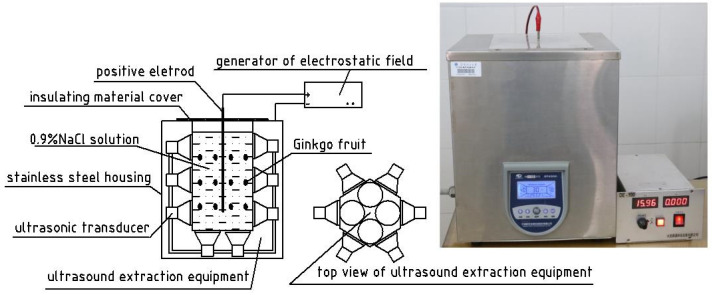
Schematic and physical diagram of the structure of the electrostatic field–ultrasonic coupling device.

Our study utilized a hexagonal ultrasound cleaning machine with a high-voltage DC power supply to create a static electricity field–ultrasound coupling device, illustrated in Figure 1. The high-voltage DC power supply was manufactured by Dalian Dingtong Technology Development Co., Ltd. Ningbo, China and can reach up to 50 kV. The hexagonal ultrasonic cleaner was designed by ourselves and commissioned from Ningbo Xinzhi Biotechnology Co., Ningbo, China. The container tank, with a base area of 234.476 cm^2^ and a height of 25 cm (volume 5861.9 cm^3^), is designed in the shape of a hexagonal cylinder. The insulated electric field electrodes are inserted into the center of the ultrasonic cleaner. The conductive electrodes are at equal distances from both the bottom and the side walls. The ultrasonic frequency is set to 40 kHz. There are a total of 18 ultrasonic transducers on the sides of the extraction tank, 3 on each side. The ultrasonic field emitted sideways is parallel to the electric field. Due to the limited area at the bottom, only 4 transducers are installed. It emits ultrasonic waves perpendicular to the electric field from the bottom. The hexagonal ultrasonic cleaning machine can directly display the ultrasonic power, the side wall ultrasonic power is adjustable from 0 to 900 W, and the bottom ultrasonic power is adjustable from 0 to 200 W. For convenience, the side wall was used to provide ultrasound in all experiments. The device enhances the ultrasonic cavitation effect by introducing the electrostatic field to improve the drying pretreatment efficiency and shorten the drying time.

We sourced ginkgo fruits from Tai’an, Jiangsu, China, which were in perfect condition, with uniform maturity, texture, shape, and color. We stored them in a refrigerator at 4 °C. For each experiment, 100.0 g ± 0.1 g of ginkgo fruits were weighed on an electronic weighing balance. To achieve better drying results, we prepared a 0.9% NaCl water solution to remove wax from the fruit surface through ultrasonic cleaning. After cleaning, we gently pressed the fruits to absorb surface moisture, weighed them on an electronic balance, and recorded the initial mass as m_1_. We then set up the hot air dryer, waited for it to reach the desired temperature, and stabilized them for 5 min. Laid the pretreated fruits in a single layer in the dryer and left them for 2 h. After removing them from the dryer, we weighed them again on an electronic balance and recorded the dried mass as m_2_. We calculated the MR using Equation (1).

(1) $$MR=\frac{M_{t}}{M_{0}}\times 100\%$$

where $M_{t}$ represents the mass of ginkgo fruits at time *t*, unit g, and $M_{0}$ represents the initial mass of ginkgo fruits, unit g.

The drying rate is calculated by Equation (2):

(2) $$R_{w}=\frac{M_{0}-M_{t}}{M_{0}t}\times 100\%=\frac{1-MR}{t}$$

where *t* is the drying time in h.

### 2.2. Experimental Methods

We first conducted single-factor experiments to determine the optimum range of parameters for ultrasonic power, pretreatment time, hot air drying temperature, and electrostatic field voltage. For each experiment, 100.0 g ± 0.1 g of ginkgo fruits was weighed and each set of experiments was repeated three times. After obtaining the optimum parameter range, the Box–Behnken method of response surface methodology was used to obtain the optimal parameters.

After obtaining the optimal conditions, in order to study the variation in MR of ginkgo fruits with time under the optimal conditions, we likewise weighed 100.0 g ± 0.1 g of ginkgo fruits per experiment. After being placed in 0.9% NaCl solution for ultrasonic cleaning, hot air drying experiments were performed. Every 30 min, we removed the fruits from the drying oven and measured their MR values. The experiment was stopped when the MR change in two consecutive ginkgo fruits was less than 2%.

Generally, the coefficient of determination (*R*^2^), the reduced chi-square(*χ*^2^), and the root mean square error (RMSE) were used to assess the goodness of fit of the tested mathematical model to the experimental data. A higher value of *R*^2^ indicates a better fit between the empirical and predicted values. lower values of *χ*^2^ and RMSE indicate better goodness of fit. The formulae for these three test indicators are as follows:

(3) $$R^{2}=1-\frac{\sum_{i=1}^{N}{\left(MR_{pre,i}-MR_{exp,i}\right)}^{2}}{\sum_{i=1}^{N}{\left(MR_{pre,i}-MR_{exp,mean}\right)}^{2}}$$

(4) $$\chi^{2}=\frac{\sum_{i=1}^{N}{\left(MR_{exp,i}-MR_{pre,i}\right)}^{2}}{N-z}$$

(5) $$RMSE=\sqrt{\frac{\sum_{i=1}^{N}{\left(MR_{pre,i}-MR_{exp,i}\right)}^{2}}{N}}$$

where $MR_{pre,i}$ is the predicted moisture radio at the ith moment, $MR_{exp,i}$ is the experimental moisture radio at the *i*th moment, *N* is the number of experimental data, and *z* is the number of constants in the model.

## 3. Results and Discussion

### 3.1. Effect of Ultrasound Power on the Moisture Content of Ginkgo Fruits

**Figure 2:**
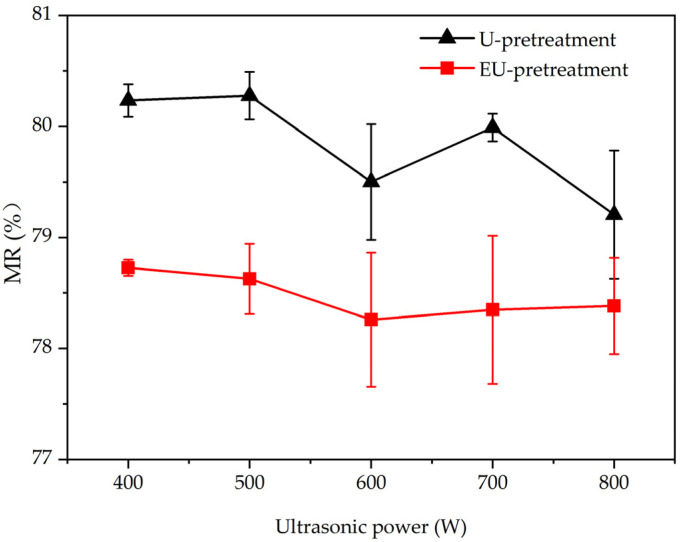
Effect of ultrasonic power on moisture of ginkgo fruits.

For EU-pretreatment, we set the electrostatic field voltage to 10 kV, the pretreatment time to 30 min, the hot air drying temperature to 75 °C, and the drying time to 2 h. U-pretreatment had the same parameters, except for the electrostatic field voltage, which was set to 0. We examined the impact of ultrasonic power (400 W, 500 W, 600 W, 700 W, 800 W) on the MR of ginkgo fruits during drying. All experiments were replicated three times and the results are presented in Figure 2.

The effect of ultrasonic electric power on the moisture of ginkgo fruits is shown in Figure 2. With the increase in ultrasound power, the MR of ginkgo fruits fluctuated in the range of 78.5–80.5%. Therefore, under the experimental conditions, the influence of ultrasonic electric power on the MR of ginkgo fruits is not obvious. However, under the comparison of EU-pretreatment and U-pretreatment, the average drying rate of EU-pretreatment increased by 0.686%. The lowest MR of ginkgo fruits was 78.26% at 600 W, indicating that the optimal ultrasonic power for EU-pretreatment was between 500~700 W. Although there was an increasing trend of water content at 700 W, it was due to the fact that ultrasound mainly changed the microstructure of ginkgo fruits [12], and the different structures of ginkgo fruits themselves led to a large error line in water content during the experiment.

### 3.2. Effect of Pretreatment Time on the Moisture Content of Ginkgo Fruits

**Figure 3:**
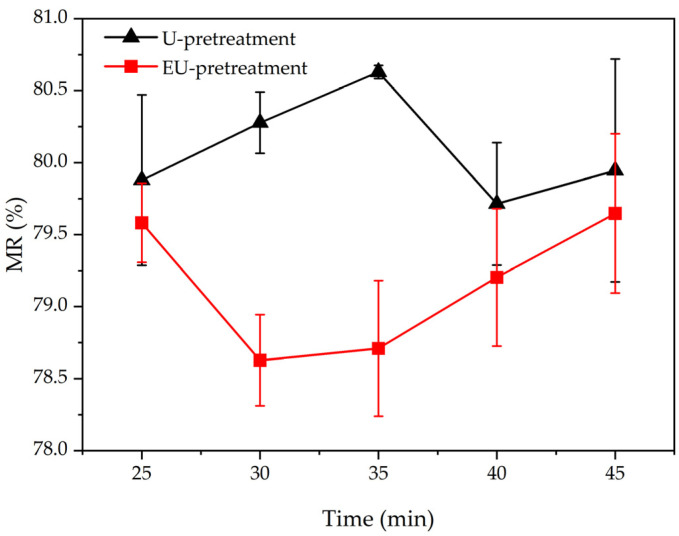
Effect of pretreatment time on moisture of Ginkgo fruits.

We conducted experiments to investigate the effect of pretreatment on the MR of ginkgo fruits during drying. Two pretreatment methods were used: EU-pretreatment and U-pretreatment. During EU-pretreatment, we set the static field voltage to 10 kV, ultrasonic power to 700 W, hot air drying temperature to 75 °C, and drying time to 2 h. For U-pretreatment, the static field voltage was 0, while the other parameters remained the same. We varied the pretreatment time between 20 and 40 min and repeated all experiments three times. The results are presented in Figure 3.

The effect of pretreatment time on the moisture of ginkgo fruits is shown in Figure 3. EU-pretreatment and U-pretreatment changed in different trends. Specifically, during EU-pretreatment, the MR values of ginkgo fruits showed a decreasing trend followed by an increasing trend, while the opposite was true for U-pretreatment. The average drying rate of EU-pretreatment increased by 0.468%. The lowest MR value of 75.26% was reached after 30 min of pretreatment, indicating that the optimal pretreatment time is between 25–35min. The trend of MR changes for the U-pretreatment was due to the longer soaking time of the ginkgo fruits in water prior to the 35 min pretreatment, which resulted in higher initial MR values and drying became more difficult. Beyond 35 min, the external water solution diffused through the stem tissue, causing the MR to reach a saturated state. As we extended the pretreatment time, the ultrasonic “wall-breaking” effect became more pronounced, leading to local rupture of the cell membrane, and opening up most of the microchannels in the ginkgo fruits. This transfer of soluble solids and water within the fruits accelerates the drying rate. At 45 min, we observed a slight increase in MR, possibly due to the sponge-like structure formed by long-term compression and stretching under ultrasound, which partially overlapped and blocked some microchannels, affecting drying efficiency to some extent.

### 3.3. Effect of Hot Air Drying Temperature on the Moisture Content of Ginkgo Fruits

**Figure 4:**
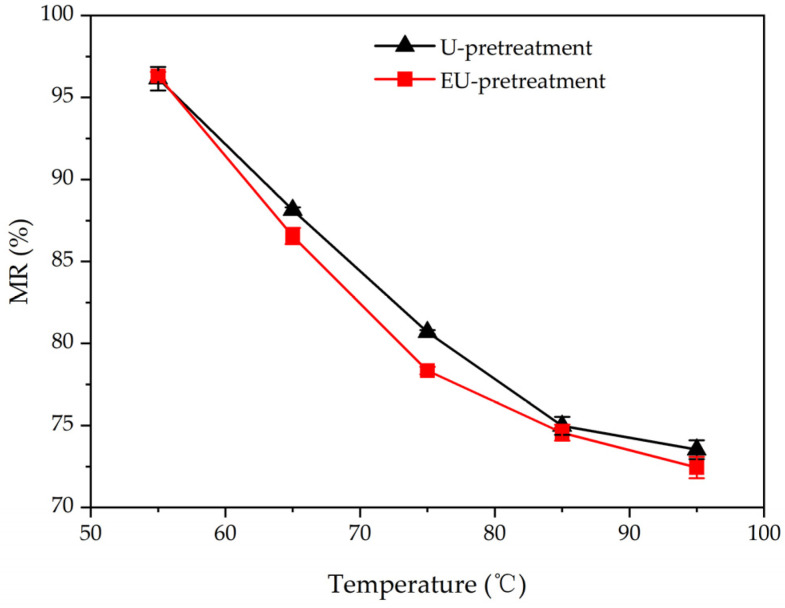
Effect of hot air drying temperature on moisture of Ginkgo fruits.

In our study, we investigated the effect of electric field coupled ultrasound preprocessing and ultrasound preprocessing on the drying of ginkgo fruits. When using EU-pretreatment, we set the electric field voltage to 10 kV, ultrasound power to 700 W, preprocessing time to 30 min, and hot air drying time to 2 h. For U-pretreatment, the electric field voltage was set to 0, and all other parameters were kept constant. We tested the impact of various hot air drying temperatures (55 °C, 65 °C, 75 °C, 85 °C, and 95 °C) on the MR of the dried ginkgo fruits. Each experiment was repeated three times, and we present the results in Figure 4.

The results in Figure 4 show that the hot air temperature has a significant effect on MR. With increasing temperature, both EU-pretreatment and U-pretreatment showed a decreasing trend; between 65 °C and 85 °C, EU-pretreatment gave better results than U-pretreatment, and the average drying rate of EU-pretreatment was increased by 0.527%. Lu et al.’s research explains that surface evaporation and the diffusion of moisture inside the food are the main mechanisms of hot air drying [13]. High temperatures can quickly promote the transfer of water from the interior to the surface, resulting in a decrease in MR. The results of this study showed that the optimal drying temperature of ginkgo fruits should be below 85 °C, as the fruit already showed a roasted state when dried for two hours at 95 °C.

### 3.4. Effect of Electric Field Voltage on the Moisture Content of Ginkgo Fruits

**Figure 5:**
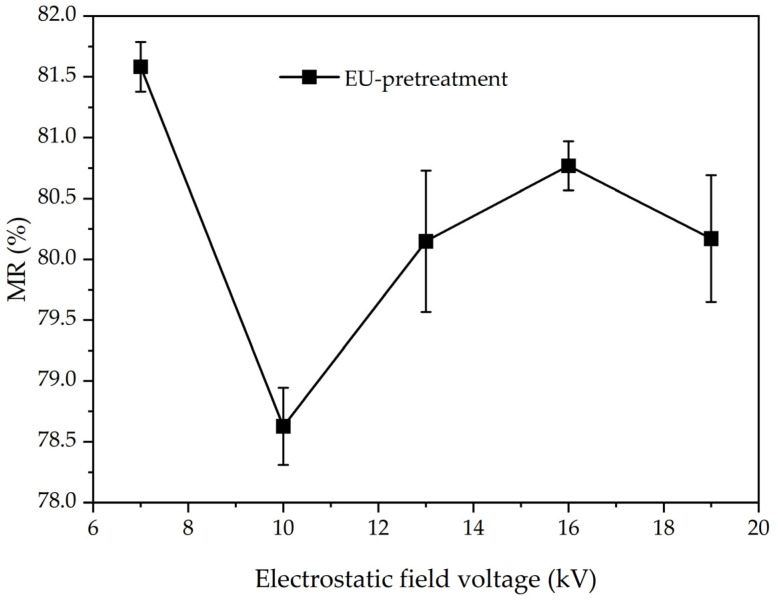
Effect of electrostatic field voltage on moisture of Ginkgo fruits.

For EU-pretreatment, the ultrasonic power and pretreatment time were set at 700 W and 30 min, respectively, while hot air was used to dry the ginkgo fruits at 75 °C for 2 h. In contrast, U-pretreatment involved a zero electric field voltage and the same parameters as the EU-pretreatment. We investigated the effect of different electric field voltages (7 kV, 10 kV, 13 kV, 16 kV, 19 kV) on the MR of the fruits. All experiments were conducted in triplicate, and the results are presented in Figure 5.

Figure 5 shows that the MR of ginkgo fruits generally decreases and then increases with increasing electrostatic field voltage. The best drying effect is observed at 10 kV, where the MR reaches a minimum of 78%. We continued to increase the voltage and the MR no longer increased. The main reason for this is that ultrasound and high-voltage electrostatic fields have a destructive effect on cell walls and membranes. An external electric field is applied to the cell membrane of ginkgo fruits to produce a potential, at which point the cell membrane can be viewed as a capacitor filled with a low dielectric constant material. When exposed to an electric field, the ions inside the cell move along the field, causing charge separation in the cell membrane. If the transmembrane potential exceeds a critical value, the repulsive force between the charged molecules causes pores to form on the cell membrane, allowing liquid and substances to flow out of the cell [14]. However, as the electric field voltage increases, the MR tends to approach a stable value and increasing the voltage does not significantly affect the MR.

### 3.5. Optimization of Process Parameters Using Response Surface Methodology

#### 3.5.1. Variable Values and Experimental Design

In this single-factor experiment, we determined the response variables to be ultrasound power, pretreatment time, hot air drying temperature, and electrostatic voltage, with each experiment having three levels (−1, 0, and +1). We denoted hot air drying temperature, electrostatic field voltage, pretreatment time, and ultrasound power as *X*_1_, *X*_2_, *X*_3_, and *X*_4_, respectively, based on their degree of influence on the experiment. Table 1 presents the levels of the input variables in both coded and uncoded forms. Using the Box–Behnken design response surface [15], we conducted 29 experiments and present the factor and level arrangement and experimental results in Table 2. We also tested the model’s fit using variance analysis and reported the results in Table 3. Our findings provide valuable insights into the impact of these variables on the experiment, and we believe this study has important implications for future research in this area.

#### 3.5.2. Equation Fitting and Variance Analysis

We conducted quadratic polynomial regression analysis on the experimental data on Design-Expert 10.0 software to determine the regression coefficients for the linear terms, quadratic terms, and interaction terms. A quadratic polynomial equation is derived through the regression analysis as follows:

(6) $$\begin{array}{cc} MR & =+0.7839469-0.0611514438X_{1}-9.64005375\times 10^{-3}X_{2}\\ & +3.31414125\times 10^{-3}X_{3}-2.429152\times 10^{-3}X_{4}+2.85774125\times 10^{-3}X_{1}X_{2}\\ & +6.3684525\times 10^{-4}X_{1}X_{3}-6.389678\times 10^{-3}X_{1}X_{4}-7.38759\times 10^{-4}X_{2}X_{3}\\ & +1.9205065\times 10^{-3}X_{2}X_{4}-2.84996\times 10^{-3}X_{3}X_{4}+0.02206443X_{1}^{2}\\ & +6.88918251\times 10^{-3}X_{2}^{2}+0.01052024X_{3}^{2}+7.0587919\times 10^{-3}X_{4}^{2} \end{array}$$

In the equation, *X*_1_, *X*_2_, *X*_3_, and *X*_4_ represent the codes of four influencing factors, including drying temperature, electrostatic field voltage, pretreatment time, and ultrasonic power, respectively.

The results in Table 3 indicate that the experimental model fits well with the experimental results, as shown by high F values and very low probabilities (Pr > F < 0.05). The analysis of variance shows that the linear and quadratic terms of the polynomial model and most interaction terms are highly significant (Pr > F < 0.05). For instance, the hot air drying temperature (*X*_1_), electrostatic field voltage (*X*_2_), and quadratic terms *X*_1_^2^ and *X*_3_^2^ significantly affect the MR of ginkgo fruits. The interaction terms also reveal significant interactions between the four factors. For example, the interaction between hot air drying temperature (*X*_1_) and preprocessing time (*X*_4_) suggests a correlation between the two, with preprocessing time affecting the MR inside the ginkgo fruits, which in turn affects the MR during hot air drying. However, there is no significant correlation between *X*_1_*X*_2_, *X*_1_*X*_3_, *X*_1_*X*_4_, *X*_2_*X*_3_, *X*_2_*X*_4_, and *X*_3_*X*_4_. The determination coefficient is relatively high at 97.52%, with a corrected determination coefficient *R*^2^ = 95.04% indicating a good fit between the experimental model and results [16]. The significant differences in independent factors are notable. The coefficient of variation (*CV*) is relatively low at 1.9%, suggesting high reliability and accuracy in the experiment.

#### 3.5.3. Response Surface Analysis

**Figure 6:**
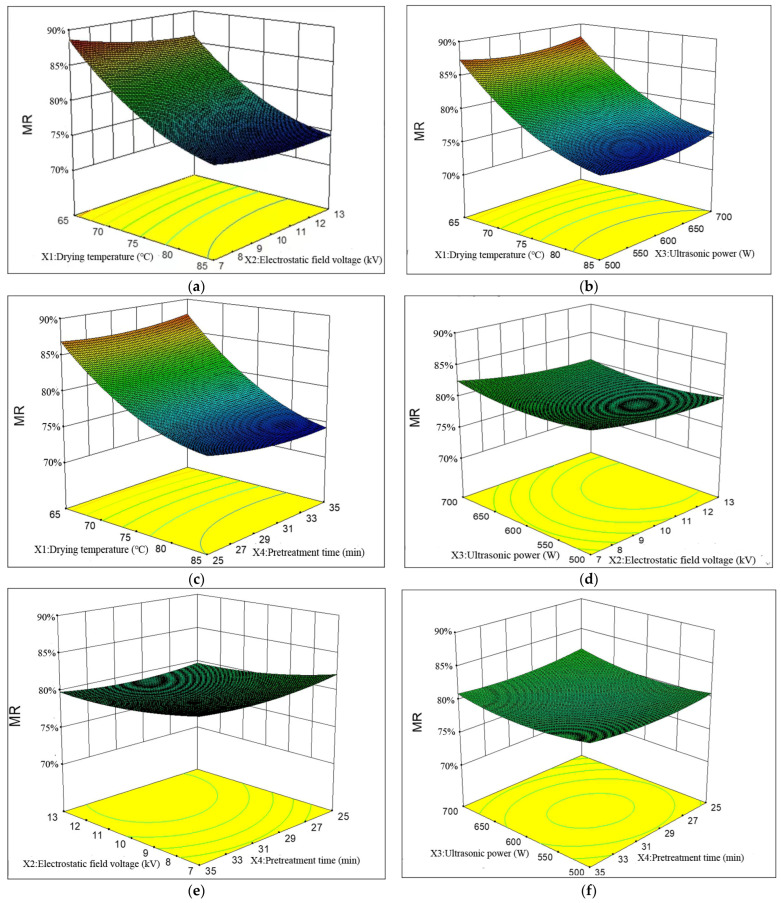
Three-dimensional response surface diagram of effects of dryingfactors on moisture content of ginkgo fruits: (**a**) hot air drying temperature and electric field voltage, (**b**) hot air drying temperature and ultrasonic power, (**c**) hot air drying temperature and pretreatment time, (**d**) electric field voltage and ultrasonic power, (**e**) electric field voltage and pretreatment time, (**f**) ultrasonic power and pretreatment time.

To predict the relationship between the independent factor and the response, we kept the other two variables fixed and constructed a three-dimensional surface response graph [17]. Figure 6 displays the MR of various factors as a 3D surface and contour map.

The model predicts that the optimal process conditions for drying ginkgo fruits are an electrostatic field voltage of 11.252 kV, an ultrasonic power of 590.074 W, a processing time of 32.799 min, and a hot air drying temperature of 85 °C. These conditions are expected to yield a minimum MR of 74.0502% after 2 h of drying.

To validate the results, three experiments were conducted at the optimal conditions using 100.0 g ± 0.1 g of ginkgo fruits. The optimal MRs obtained from the experiments were 74.456%, 74.858%, and 74.60%, respectively, with an average value of 74.64% and a relative error of 0.187%. These results demonstrate the effectiveness of the proposed method.

### 3.6. Ginkgo Fruits’ Moisture Content Drying Model

After the EU-pretreatment, we conducted drying experiments on them under approximately optimal conditions based on the common mathematical model of the drying curve and fitted using origin pro 2016. We calculated these three values of *R*^2^, *χ*^2^, and RMSE to test the accuracy of the model fit. The statistical results of the treated ginkgo fruit drying model are shown in Table 4, and the fitted curves are shown in Figure 6.

After analyzing the fitting results and data, the Page, logarithmic, Midilli, two-term, and cubic models were found to have a good fit to the actual MR of the experiment, with determination coefficients above 0.9. Among them, the two-term model was found to be the most suitable for this drying experiment, with a high fit and a determination coefficient of 0.99965.

**Figure 7:**
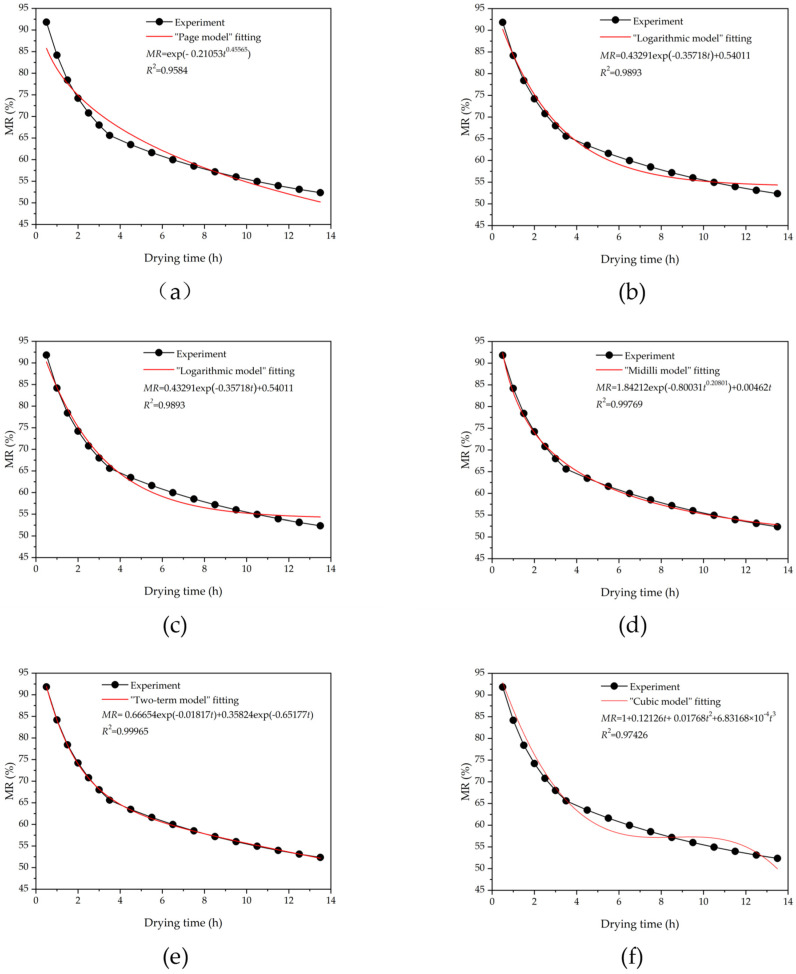
The drying curves fitted by several common models: (**a**) Page model, (**b**) Henderson and Pabic model, (**c**) Logarithmic model, (**d**) Midilli model, (**e**) Two−term model, (**f**) Cubic model.

As shown in Figure 7e, the MR of Ginkgo fruits decreased steadily during the drying experiment, starting with a steep decline and gradually slowing down. The reduction in MR was attributed to the rapid increase in surface temperature, leading to an increase in water vapor pressure inside the fruit, which in turn broke down the cell structure. Additionally, the pretreatment with ultrasound and an electric field opened the microchannels of the cells in ginkgo fruits, resulting in a faster drying rate. The two-term model predicted the MR of the fruit with a high degree of accuracy, further proving its effectiveness in predicting the drying process of ginkgo fruits.

## 4. Conclusions

(1) An electric field significantly improves the efficiency of the hot air drying of ginkgo fruits compared to ultrasonic and electrostatic–ultrasound pretreatment. Single-factor experiments showed that factors such as ultrasonic power, ultrasonic electric field pretreatment time, hot air drying temperature, and electrostatic field voltage influence the drying rate.
(2) Using the Box–Behnken design in response surface methodology, the optimal parameters for drying ginkgo fruits to the desired MR were obtained. These parameters were an electrostatic field voltage of 11.252 kV, an ultrasonic power of 590.074 W, a pretreatment time of 32.799 min, and a hot air drying temperature of 85 °C. The predicted MR after 2 h of hot air drying was 74.0502%, which was experimentally verified with a relative error of only 0.187%. The experimentally obtained values were also in good agreement with the predicted values.
(3) The MR of ginkgo fruits under optimal conditions was plotted against time to create an MR-t chart, and the fitting degree of seven models describing the drying process of ginkgo fruits was analyzed. Among these models, the Page model, logarithmic model, Midilli model, two-term model, cubic model, and experimentally obtained values had a determination coefficient above 0.9. The two-term model was the most suitable model for this drying experiment with a high degree of fitting and a determination coefficient of 0.99965.

EU-pretreatment can become an important development trend in dying pretreatment technology. In order to advance the theory and time of electric-field-enhanced ultrasonic drying, the relationship function and model of electrostatic field and ultrasonic coupling energy can be explored through theoretical calculations to provide a scientific basis for knowledge-based drying calculations and dryer design. Future modeling of moisture-content-based volume shrinkage coefficients, moisture diffusion coefficients, rehydration rates, and coloration of dried products can also be undertaken in the hope of obtaining a reasonably accurate prediction equation. In addition, electric-field-assisted drying technology is an important research direction, and the next research hopes to obtain the effect of an enhanced drying rate by using ultrasonic synergistic hot air or electrostatic field synergistic hot air [24].

## Figures and Tables

**Table 1:** Coded and uncoded values of variables and their levels.

Factor	Coding	Unit	Levels		
			−1	0	1
Drying temperature	*X*_1_	°C	65	75	85
Electrostatic field voltage	*X*_2_	kV	7	10	13
Ultrasonic power	*X*_3_	W	500	600	700
Pretreatment time	*X*_4_	min	25	30	35

**Table 2:** Factors and levels of design and layout of response surface center composite experimental results.

Runs	Drying Temperature (°C)	Electrostatic Field Voltage (kV)	Ultrasonic Power (W)	Pretreatment Time (min)	MR (%)
1	65	10	600	25	87.28
2	85	10	700	30	76.4
3	85	10	600	25	75.98
4	75	7	700	30	81.18
5	85	10	600	35	74.89
6	75	7	600	35	78.93
7	75	10	600	30	81.53
8	65	13	600	30	85.44
9	75	13	500	30	80.08
10	75	7	500	30	83.99
11	75	10	500	35	80.38
12	75	10	700	25	80.47
13	65	10	500	30	86.37
14	75	10	600	30	78.08
15	85	7	600	30	76.54
16	75	10	600	30	79.71
17	75	10	600	30	78.36
18	75	10	600	30	77.61
19	85	10	500	30	74.05
20	65	7	600	30	89.03
21	75	13	600	35	78
22	75	7	600	25	81.15
23	85	13	600	30	74.1
24	65	10	700	30	88.31
25	75	13	700	30	79.58
26	75	10	500	25	79.62
27	75	10	700	35	80.09
28	65	10	600	35	89.38
29	75	13	600	25	79.45

**Table 3:** ANOVA for response surface quadratic model.

Source	Sum of Squares	df	Mean Square	F Value	*p*-Value Prob > F	Significance
Model	0.050	14	3.565 × 10^−3^	39.34	<0.0001	significant
*X*_1_	0.050	1	0.045	495.19	<0.0001	**
*X*_2_	0.045	1	1.115 × 10^−3^	12.31	0.0035	**
*X*_3_	1.115 × 10^−3^	1	1.318 × 10^−4^	1.45	0.2478	
*X*_4_	1.318 × 10^−4^	1	7.081× 10^−5^	0.78	0.3916	
*X*_1_ *X*_2_	7.081 × 10^−5^	1	3.267× 10^−5^	0.36	0.5578	
*X*_1_ *X*_3_	3.267 × 10^−5^	1	1.622 × 10^−6^	0.018	0.8955	
*X*_1_ *X*_4_	1.622 × 10^−6^	1	1.633 × 10^−4^	1.80	0.2008	
*X*_2_ *X*_3_	1.633 × 10^−4^	1	2.183 × 10^−6^	0.024	0.8789	
*X*_2_ *X*_4_	2.183 × 10^−6^	1	1.475 × 10^−5^	0.16	0.6927	
*X*_3_ *X*_4_	1.475 × 10^−5^	1	3.249 × 10^−5^	0.36	0.5589	
*X*_1_^2^	3.249 × 10^−5^	1	3.158× 10^−3^	34.85	<0.0001	**
*X*_2_^2^	3.158 × 10^−3^	1	3.079 × 10^−4^	3.40	0.0866	
*X*_3_^2^	3.079 × 10^−4^	1	7.179 × 10^−4^	7.92	0.0138	*
*X*_4_^2^	7.179 × 10^−4^	1	3.232 × 10^−4^	3.57	0.0799	
Residual	3.232 × 10^−4^	14	9.062 × 10^−5^			
Lack of Fit	1.269× 10^−3^	10	1.062 × 10^−4^	2.05	0.2546	not significant
Pure Error	1.062× 10^−3^	4	5.173 × 10^−5^			
Cor Total	2.069 × 10^−4^	28				
Total: *R*^2^ = 97.52% Adj-*R*^2^ = 95.04% *CV* = 1.9%						

**: There was a significant difference (*p* < 0.01); *: The difference was significant (*p* < 0.05).

**Table 4:** Mathematical models of common drying curve.

No.	Drying Model	Model Equation	*R* ^2^	*χ* ^2^	RMSE
1	Page [18]	$MR=\exp(-kt^{n})$	0.9584	5.9379 × 10^−5^	0.043891578
2	Henderson and Pabic [19]	MR=a×exp⁡(−kt)	0.84707	0.00218	0.03275
3	Logarithmic [20]	MR=a×exp⁡(−kt)+b	0.9893	1.63696 × 10^−4^	0.011606286
4	Midilli [21]	$MR=a\times \exp(-kt^{n})+bt$	0.99769	3.8042 × 10^−4^	0.005393596
5	Two-term [22]	$MR=a\times \exp(-k_{1}t)+b\times \exp(-k_{2}t)$	0.99965	5.78947 × 10^−6^	0.002104101
6	Cubic [23]	$MR=1+at+bt^{2}+ct^{3}$	0.97426	0.00293	0.050880483

## Data Availability

Data are contained within the article.
